# Effective treatment of severe stenosis of the carotid and coronary arteries: a case study

**DOI:** 10.1186/s13019-023-02135-2

**Published:** 2023-01-18

**Authors:** Kayo Sugiyama, Fuminori Ato, Hirotaka Watanuki, Masato Tochii, Shigeru Miyachi, Katsuhiko Matsuyama

**Affiliations:** 1 Department of Cardiac Surgery, Aichi Medical University Hospital, 1-1 Yazako Karimata, Nagakute, Aichi 480-1195 Japan; 2 Department of Neurosurgery, Aichi Medical University Hospital, 1-1 Yazako Karimata, Nagakute, Aichi 480-1195 Japan

**Keywords:** Carotid artery stenting, Coronary artery bypass grafting, Arterial spin labeling, Diffusion-weighted image, Diffusion-perfusion mismatch

## Abstract

It is considered acceptable to conservatively manage coronary artery bypass grafting patients with carotid artery disease without the need for preoperative corrective carotid revascularization. However, in the present case, rapidly progressive stroke symptoms with penumbra suggested in the arterial spin labeling, carotid artery stenting was performed successfully.

## Background

Despite improvements in anesthesia and surgical techniques, stroke remains a primary concern during assessment of cardiopulmonary bypass candidacy and a devastating neurologic complication of myocardial revascularization when bypass is performed [1]. However, there is ongoing controversy regarding management of and surgical interventions for patients with occlusive disease of the carotid arteries concomitant with heart disease [2–5]. A staged approach has been proposed, but the increased risks of myocardial infarction and bleeding as a result of dual anti-platelet aggregation therapy in the interval following carotid artery stenting (CAS) may present a limitation [6–8]. Further, in patients undergoing coronary artery bypass grafting (CABG), cardioembolism and intracranial arterial stenosis or small vessel disease are often the primary mechanisms of stroke, and thus preoperative cervical carotid revascularization would not be effective for prevention [9].

Magnetic resonance imaging (MRI) has significantly higher sensitivity and specificity than computed tomography for the diagnosis of acute ischemic infarction in the first few hours following onset [10]. The earliest rescue of the penumbra, the reversibly injured brain tissue surrounding an ischemic core and target for the treatment of acute stroke, is key for effective treatment [11]. Arterial spin labeling (ASL) is an alternative non-invasive perfusion method that does not require contrast and qualitative estimation of the perfusion- diffusion-weighted image mismatch using ASL as the perfusion method could be possible [12] through which the penumbra can be speculated [13].

Herein, we successfully treated a patient with CAS who presented with acute cerebral infarction following CABG. The mismatch between diffusion-weighted image (DWI) and ASL was useful for detecting the penumbra. Debate exists as to whether a detailed perfusion evaluation should be performed preoperatively along with imaging evaluation for marginal cases of severe carotid artery disease complicated by severe coronary artery disease [9, 14]. If symptoms worsen rapidly, immediate revascularization may result in a good recovery; however, a hybrid approach in which coronary artery bypass grafting is performed immediately following carotid revascularization can also be effective, and therefore should be considered for these marginal lesions [15–17]

## Case presentation

**Fig. 1:**
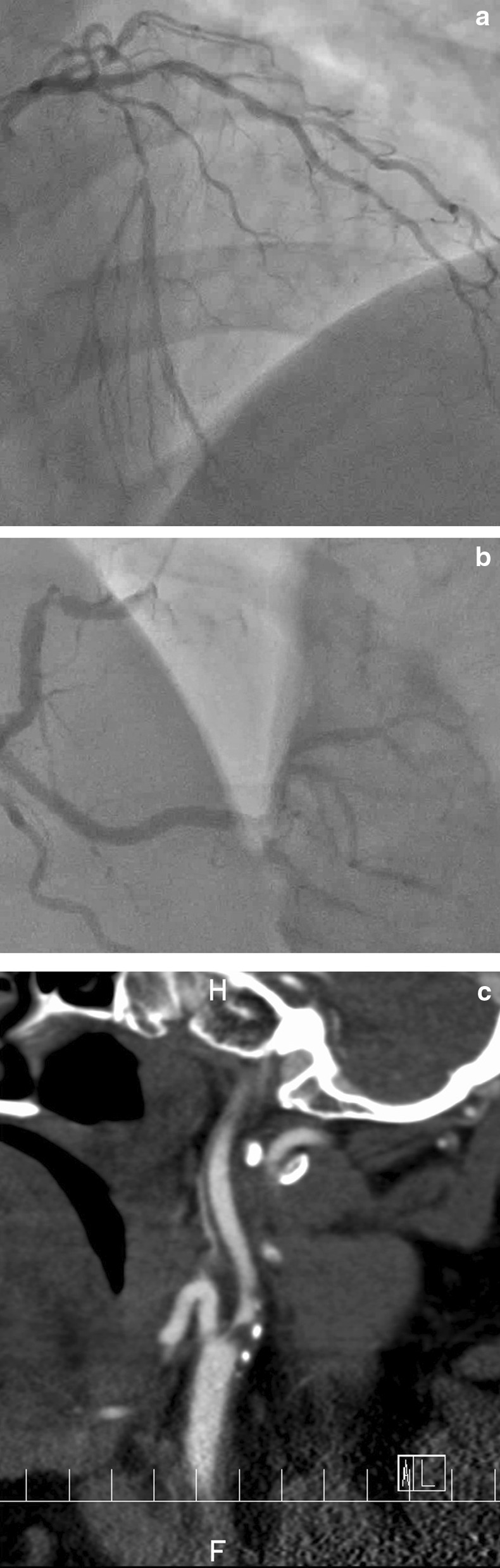
**a** Preoperative left coronary angiography showing severe stenosis of the left circumflex and left anterior descending arteries. **b** Preoperative right coronary angiography showing severe stenosis of the right coronary artery. **c** Preoperative computed tomography scan showing severe stenosis of the left common carotid artery (white arrowhead)

A 45-year-old man with chest discomfort on effort was referred to our hospital. He had been treated for hypertension, hyperlipidemia, hyperuricemia, and diabetes mellitus for 5 years. He had a current smoking habit and a body mass index of 35 indicating obesity, and no family history. Coronary angiography demonstrated severe coronary artery disease including severe stenosis of the left anterior descending artery, the diagonal branch, and the right coronary artery, as well as total occlusion of the left circumflex artery (Fig. 1A, 1B). Further examinations also revealed severe stenosis of the left internal carotid artery without any neurological symptoms (Fig. 1C). Because this was a marginal case for carotid artery revascularization, discussion was held to discern between the neurosurgery and cardiac surgery departments, with neurosurgeons suggesting that preemptive carotid artery stenting was not necessary. They recommended that blood pressure and hemoglobin levels be maintained during CABG, and that antiplatelet therapy be started as soon as possible following the procedure.

Following a median sternotomy, the left internal mammary artery was harvested, followed by harvest of a segment of the saphenous vein using a skip skin incision. On-pump beating CABG was then performed using the left internal mammary artery to the left anterior descending artery, one saphenous vein graft to the diagonal branch and the posterior lateral branch with the sequential fashion, and another saphenous vein graft to the proximal and distal site of the right coronary artery. During the procedures, mean blood pressure was maintained above 60 mmHg with continuous monitoring of cerebral oxygen saturation, which showed no significant decrease in bilateral oxygen levels. Because the bleeding was minimal and did not cause anemia, no blood transfusion was required.

**Fig. 2:**
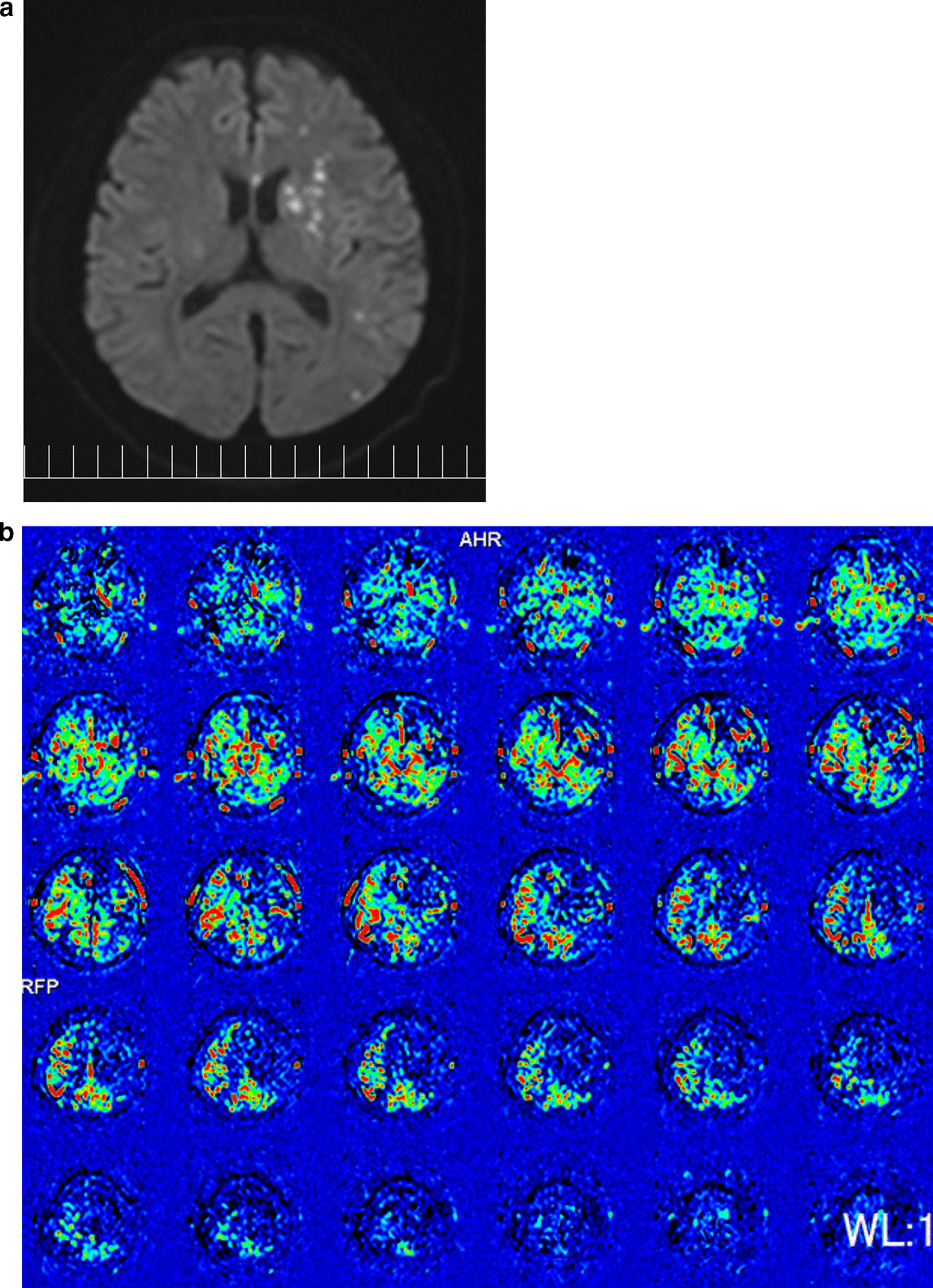
**a** Postoperative diffusion-weighted image of the magnetic resonance imaging showing multiple high intensity areas in the left lobe. **b** Postoperative arterial spin labeling of the magnetic resonance imaging showing low perfusion areas in the left lobe, detecting penumbra

The patient tolerated the procedure well and had an uncomplicated postoperative course with no neurological deficits initially. The day following CABG, antiplatelet therapy was begun. However, on the morning of the second day, the patient developed a sudden speech impediment and paralysis of the right side of his body. The patient’s National Institutes of Health Stroke Scale was as high as 14. Because he had appeared to sleep well through the night, the stroke might have begun within the preceding eight hours. An urgent brain MRI was performed to evaluate cerebral ischemia and revealed a subacute cerebral infarction in the left lobe (Fig. 2A) and MR angiography revealed almost occlusion in the left common carotid artery. This was followed by ASL showing a more extensive zone of low perfusion in the same area, and there was a mismatch between DWI (Fig. 2A) and ASL (Fig. 2B) indicating a penumbra. Perfusion-weighted image was considered difficult to perform due to progression of renal dysfunction. Because of the rapid deterioration of the patient’s neurological symptoms over time and the significance of saving the penumbra, the decision was made to perform an emergency revascularization. Thus, neurosurgeons performed emergency CAS for severe stenosis of the left internal carotid artery.

**Fig. 3:**
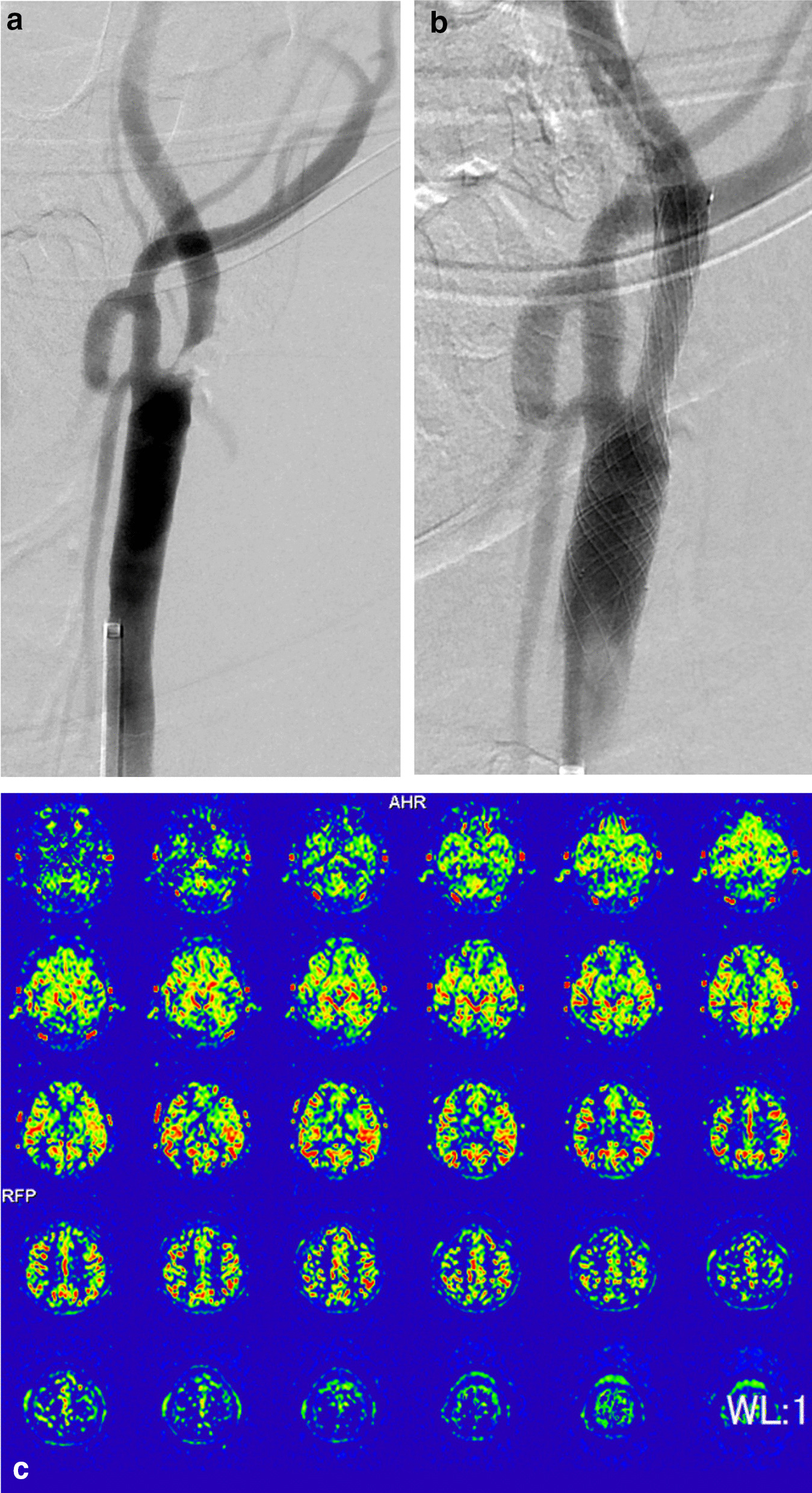
**a** Preprocedural carotid angiography showing severe stenosis of the left common carotid artery. **b** Postprocedural carotid angiography showing improvement of the severe stenosis of the left common carotid artery. **c** Postprocedural arterial spin labeling of the magnetic resonance imaging showing improvement of the low perfusion areas in the left lobe

Stenting to the left internal carotid artery was successfully performed with local anesthesia. Percutaneous access to the carotid artery was obtained from a common femoral artery using an 8Fr sheath. Lesions were crossed with a 0.035-inch, angle, and hydrophilic guidewire with a supporting 8Fr straight catheter. Carotid artery angiography showed almost occlusion in the left common carotid artery (Fig. 3A). Preprocedural ballooning with distal blocking catheter (OPTIMO EPD balloon guide catheter,™ Tokai Medical Products, Aichi, Japan) was performed, and a 10 × 20 mm balloon expandable stent (CASPER,™ Terumo, NJ, USA) was placed with postprocedural ballooning. The systemic blood pressure and INVOS dropped by 30 mmHg degrees and 10% during the procedure, with both monitors recovering promptly. A small fresh thrombus was removed via the aspiration catheter and postoperative angiography confirmed improvement of the stenosis (Fig. 3B). The carotid plaque at the bifurcation may have caused regional hypoperfusion or act as an embolic source in causing a stroke. Strict treatment with dual antiplatelet agents was administered beginning the day following carotid artery stenting. ASL following the procedure revealed improvement (Fig. 3C), and the patient’s neurological symptoms improved dramatically over the next few days. After sufficient rehabilitation, he was discharged without paralysis on postoperative day 16. After a few months of required outpatient speech rehabilitation, he then recovered to nearly normal neurological activity. At 1-year follow-up, the patient remained stable without any major adverse cerebral or cardiovascular events.

## Discussion

This study described the case of a patient with acute cerebral infarction after CABG, for whom CAS was successful performed. Despite improvements in anesthesia and surgical techniques, stroke remains a devastating neurologic complication of myocardial revascularization and a primary concern in assessing a patient’s cardiopulmonary bypass candidacy. [1] The prevalence of severe carotid artery stenosis (> 80% stenosis) among patients undergoing coronary artery bypass surgery has been estimated to be between 6 and 14% [2]. However, some reports indicate that severe carotid artery stenosis alone is not a risk factor for stroke or mortality in patients undergoing CABG [4, 5].

In a staged approach (CAS or carotid endarterectomy followed by CABG after several weeks), the increased risk of myocardial infarction in the interval may represent a limitation. Moreover, the need for dual anti-platelet aggregation therapy for 3 to 4 weeks following CAS increases the risk of bleeding if surgery is urgently required in the meantime [6]. Further, scattered reports suggest that a staged approach does not improve prognosis and may even increase risk [7, 8]. Simultaneous hybrid revascularization by CAS or carotid endarterectomy and CABG may be a viable alternative to the staged combination, particularly among patients for whom CABG cannot be postponed [15–17]. If a staged approach had been administrated in the present case, the patient might have developed a coronary attack in the interval. Considering that the carotid lesion worsened immediately following the coronary artery bypass procedure, simultaneous hybrid revascularization could have been considered.

The mechanism of perioperative stroke is still unclear, although calcific debris from a diseased valve, macroemboli of cardiac origin, introduction of air during the procedure, hypoperfusion arising from a severely stenotic carotid artery, or embolization from an ulcerated plaque have all been described in the literature [18]. Moreover, since the primary causes of stroke during cardiac surgery have been thought to be cardiogenic embolism, intracranial arterial stenosis, or small vessel disease, preoperative carotid revascularization would not have a preventive effect for these events [9]. In the present case, although there was severe stenosis of the carotid artery, it was determined that preoperative revascularization was not necessary, and the cardiac surgery was ultimately performed successfully. However, because of the potential for excessive coagulability, hypotension, and dehydration in the early postoperative stage, the carotid plaque at the bifurcation likely caused regional hypoperfusion and acted as an embolic source in the eventuation of stroke. Furthermore, in order to have predicted this phenomenon, quantitative evaluation of blood flow in the brain should have been performed preoperatively in addition to imaging evaluation.

Single-photon emission computed tomography is the most widely used method for evaluating cerebral circulatory reserve, and the rate of increase in cerebral blood flow obtained from its measurement before and after acetazolamide loading is used as an index of cerebral circulatory reserve [9]. Kuroda et al. demonstrated that cerebral blood flow changes in response to changes in blood pressure, and even asymptomatic patients may tolerate perioperative reductions in cardiac output or blood pressure poorly with increased risk of cerebral infarction [14]. However, acetazolamide has the potential to cause the serious side effect of acute pulmonary edema [19] and its use in cases with cardiac disease must be carefully evaluated. In accordance with these reports, it appears safe and feasible in most cases to conservatively manage CABG patients with severe carotid artery disease without the need for preoperative corrective carotid revascularization. However, if rapid progression of neurologic symptoms is observed postoperatively, immediate revascularization should be considered.

ASL in MRI detects perfusion without the use of exogenous contrast, instead relying on magnetic labeling of arterial blood [12]. Zaharchuk et al. reported that ASL correlates well with mismatch with DWI as a substitute for perfusion-weighed image [12]. Chalela et al. described that ASL in acute stroke successfully depicted perfusion deficits, restored perfusion, hyperperfusion, perfusion/diffusion mismatches, and delayed arterial transit in addition to providing quantitative cerebral blood flow determination [13]. The mismatch between DWI and ASL represents the penumbra, or the tissue at risk of infarction [10]. 

Although the exact timing of the stroke was also unknown in this case, the decision to perform urgent revascularization was made because of neurologic findings that rapidly progressed within the examination alone, the possibility of penumbra on imaging, and the possibility of reversible recovery with revascularization. Although immediately post-surgical cardiac patients may not be amenable to transport for MRI imaging due to the placement of various infusion pumps and lines and the limitation of hemodynamics on prolonged imaging or movement, it is recommended that these issues be worked through so that non-invasive ASL may be performed in conjunction with head MRI imaging.

## Conclusions

With prompt revascularization, we successfully treated a patient who presented with acute cerebral infarction after CABG. Multifactorial evaluation and prompt revascularization were essential for saving the penumbra, ASL could be an alternative for perfusion-weighted image.

## Data Availability

All data generated or analyzed during this study are included in this article.

## Abbreviations

ASL: Arterial spin labeling
CABG: Coronary artery bypass grafting
CAS: Carotid artery stenting
CT: Computed tomography
DWI: Diffusion-weighted image
MRI: Magnetic resonance imaging
